# Primary retroperitoneal mucinous cystadenoma: A case report with review of literature

**DOI:** 10.1016/j.amsu.2022.104818

**Published:** 2022-11-05

**Authors:** Safouane Frini, Seifeddine Ben Hammouda, Ahlem Bellalah, Chiraz Chebanne, Slim Bchir, Leila Njim, Abdelfatteh Zakhama, Rim Hadhri

**Affiliations:** a Department of Pathology, Fattouma Bourguiba University Hospital, Monastir, 5000, Tunisia; b Department of Urology, Fattouma Bourguiba University Hospital, Monastir, 5000, Tunisia; c Faculty of Medicine, University of Monastir, Monastir, 5000, Tunisia

**Keywords:** Retroperitoneal cyst, Benign retroperitoneal tumor, Mucinous cystadenoma, Primary retroperitoneal mucinous cystadenoma, Surgical pathology, Case report

## Abstract

**Introduction and importance:**

Primary Retroperitoneal mucinous cystadenoma (PRMC) is an extremely rare benign tumor, predominantly occurring in women, with unclear pathogenesis.

**Case presentation:**

A 31-year-old woman, with no medical or surgical history, presented with left flank pain.

**Clinical discussion:**

An abdominal computed tomography (CT) scan revealed an 11cm retroperitoneal cyst. Due to its large size, percutaneous CT-guided drainage followed by a laparotomy surgical resection, were performed. Post-operative course was uneventful. Histological and immunohistochemical findings were consistent with PRMC. The patient was disease-free after a 6-month follow-up.

**Conclusion:**

Mucinous cystadenoma is a very odd finding in the retroperitoneum. Multiple differential diagnoses are to be considered beforehand, as most of cystic lesions in this anatomical region are malignant and require a different surgical approach. Radical resection, by laparotomy or laparoscopy, is the treatment of choice.

## Highlights

• Primary retroperitoneal mucinous cystadenoma is a rare benign cystic tumor.
• Preoperative radiological diagnosis of benignity is fundamental.
• Histologically composed of tall columnar cells and low cuboidal cells, on a collagenous stroma.
• Surgical resection is the recommended treatment.

## 1 Introduction

Retroperitoneal cysts are a relatively common finding. They account to 0.2% of all tumors [1]. Clinicopathologically, they can be classified into three categories: benign, borderline or malignant; the last being the most frequent occurrence in this anatomical region [2]. Among the benign spectrum, primary retroperitoneal mucinous cystadenomas (PRMC) are extremely rare with, to our knowledge, fewer than 60 cases reported so far. Due to its odd location, unusual manifestations and malignancy-mimicking presentation, a proper awareness of its clinico-pathological features is important. In this report, we add yet another case of PRMC diagnosed in a 31 year-old women. This case report has been reported in line with the SCARE 2020 Criteria [3].

## 2 Case report

**Fig. 1:**
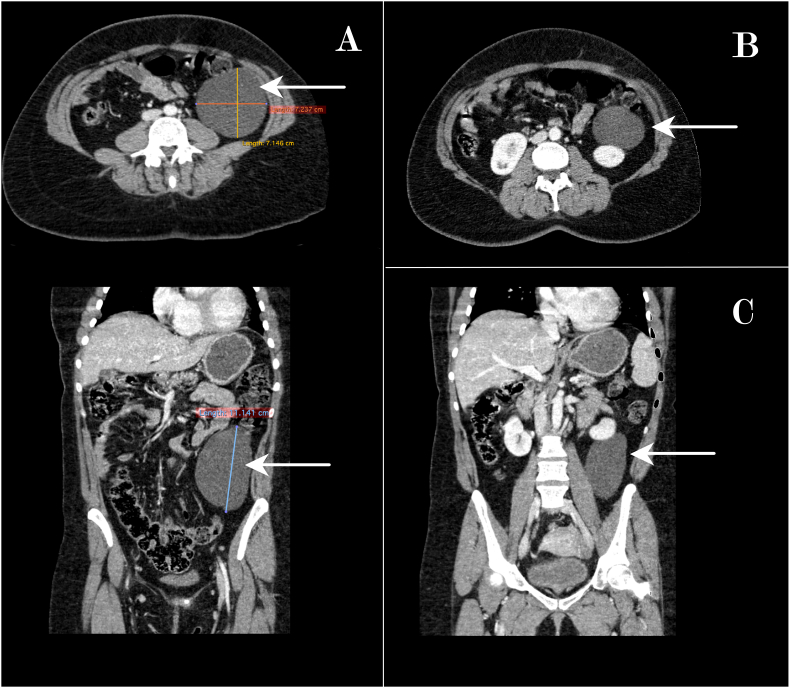
**Large retroperitoneal cystic mass:** Axial and coronal view of Abdominal computed tomography (CT) scan showing a cyst *(arrow)* with a thin wall and a homogenous liquid content and measuring 72x71x112 mm (A). The cyst exerts mass effect on the descending colon (B) and the left kidney (C).

A 31-year-old woman was admitted to the emergency department with acute lumbar pain. She had no medical or surgical history, and had not any known drug allergies. Her family history revealed hypertension, but no other significant medical or surgical diseases. The patient declared that the pain started a week before, denying her of sleep and hindering physical activity, but lately intensified and was associated with an episode of vomiting. Physical examination revealed tenderness in the left flank. Biological tests (Blood count, renal and hepatic function) were normal. A Computed tomography (CT) scan was performed and found a cystic lesion measuring 112x72x71 mm, with a thin wall and a homogenous liquid content (Fig. 1). At first, this cyst was thought to be a renal cyst, but after a second examination, it was concluded that this cyst was retroperitoneal and was independent from the kidney. The cyst exerted mass effect on the left kidney and the descending colon. Due to the resolution of her symptoms and the none-emergent nature of the situation, the patient was discharged from the Emergency ward and was given an appointment in the outpatient urology unit. One month later, she was hospitalized in the urology department of a university hospital, to receive the appropriate treatment. Eventhough the clinical and radiological presentation favored a benign lesion; malignancy could not be excluded, especially without histological confirmation. Due to the large size of the cyst and its contiguity to the left kidney and colon, it was decided to firstly perform a CT-guided percutaneous drainage of the lesion and then a radical resection by laparotomy. The drained cyst-content was a viscous clear fluid. The intervention was performed by an assistant professor, in a university hospital. Per-operative assessment showed that the cyst was located behind the posterior peritoneum of the descending colon and it had not invaded any adjacent organs. The left kidney was normal. The cyst was completely removed with no complications. The procedure was successful and went as expected. The post operative course was uneventful. The patient declared that her pain was relieved and that the symptoms had not reoccurred. The patient was discharged after 3 days of hospitalization in the urology department, during which no complications were noted. She was scheduled to have an appointment after 3 months.

**Fig. 2:**
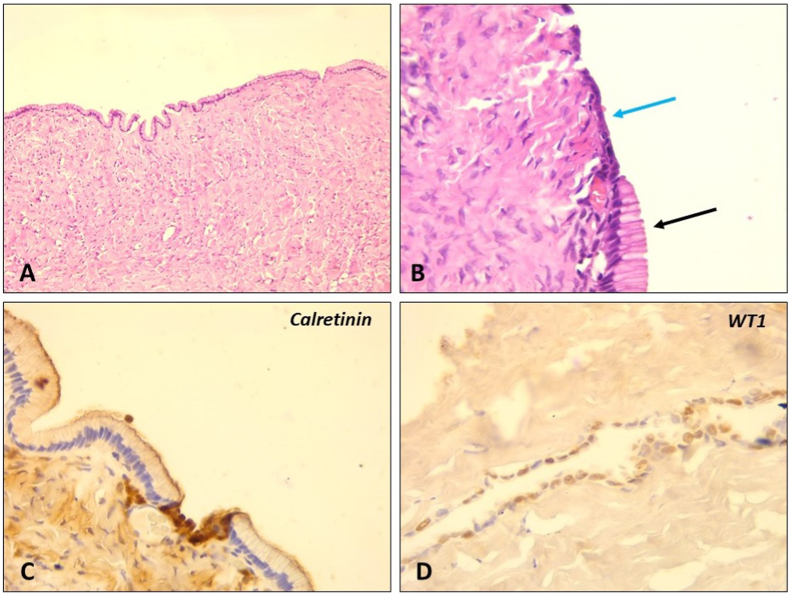
**Histological examination of the lesion:***(A*) The cyst has a collagenous and moderately cellular wall *(Scanning power). (B)* The cystic lining is composed of tall columnar cells (*black arrow*) and flat low cuboidal cells (*blue arrow*). *(High Power). (C, D)* Immunohistochemistry showed that flat low cuboidal cells expressed calretinin *(C)* and WT1 *(D),* both being mesothelial markers. . (For interpretation of the references to colour in this figure legend, the reader is referred to the Web version of this article.)

The surgical specimen was sent for pathological examination. On gross examination, the cyst had a thin wall (2mm thickness), was unilocular, without content, and measuring 70 × 68 mm. Histologically, The cystic lining was alternating between two types of cells: Tall columnar cells and flat low cuboidal cells, resembling mesothelial cells. The tall columnar cells had a clear cytoplasm charged with mucin with basal nuclei and were stained by Alcian blue. Flat low cuboidal cells had a scant cytoplasm with ovoid nuclei. There was no nuclear atypia, stratification, papillary growth or stromal invasion of epithelium. The stroma was moderately cellular and collagenous. Flat low cuboidal cells expressed, in immunohistochemistry (IHC), calretinin and N-terminus WT1 (nuclear staining) (Fig. 2).

Clinical and radiological assessments performed in the outpatient unit reveled no recurrence after a 6-month follow up.

## 3 Discussion

PRMC was first described by Hanfield-Jones in 1924 in his study on retroperitoneal cysts [4]. It is a benign proliferation that resembles pancreatic or ovarian cyst adenoma [5]. Most of the reported cases had occurred in women (95%) with age ranging from 14 to 85 (median age = 36) [2]. The etiopathogenesis of this tumor is still unknown but two majors hypothesis were recurrently discussed; the first theory states that these cysts derive from a müllerian/ovarian cell lineage. This could be explained by primordial germ cells aberrantly migrating to the retroperitoneal area, during their descent through the fetal dorsal mesentery [6]. This theory is supported by the morphological similarities with ovarian cystadenomas and the expression of the estrogen receptors (ER) on the mesothelial lining and the stroma [2]. However, it is challenged by the fact that some cases were diagnosed in male patients [7]. The second hypothesis is that PRMC derives from multipotent mesothelial cells that underwent a mucinous metaplasia [8]. This is particularly relevant, considering that flat low cuboidal cells, as displayed in our case, expressed mesothelial cell markers [9].

Clinically, patients with PRMC usually describe abdominal or pelvic pain, abdominal distention or discomfort, and in some instances, complain of a painless abdominal mass [8]. Incidental finding is also possible and the patient may remain asymptomatic, seeing that the symptoms depend on the size and the location of the cyst [10]. Large cysts could induce more severe symptoms and have a higher probability of causing complications such as rupture or infections [10]. In our case, the cyst displaced both the kidney and the colon, and could have caused intestinal obstruction or obstructive nephropathy, if it was left untreated. In previous reported cases, PRMC's size ranged from 6 to 30 cm in its largest dimension [2]. Even though retroperitoneal cysts seem to enlarge over time, one case reported a radiological assessment of an untreated retroperitoneal cyst, in 34-year-old women that shrunk from 16 × 8 cm to 10 × 4 cm over the course of 6 years. However, at the age of 41, its size increased to 17 × 14 cm and displayed three mural nodules. After surgery and histological examination of that cyst, the mural nodules showed focal microinvasive adenocarcinoma [11]. Therefore, even if mucinous cystadenomas are benign, they could potentially degenerate into a malignant tumor, if left untreated, regardless of its size.

From a clinicopathological approach, PRMC could be classified intro three subtypes: mucinous cystadenoma (≈36%), mucinous cystic tumor of borderline malignancy (≈13%), and mucinous cystadenocarcinoma (≈51%) [2]. Mixed serous and mucinous cystadenomas have also been described [12].

Histological examination of PRMC shows that the lining of the cyst alternates between two types of cells, tall columnar cells (TCC) and flat low cuboidal cells (LCC). LCC are usually positive for mesothelial markers such calretinin, cytokeratin CK 5/6, CA 125, and D2-40 and are usually negative for mucinous cell makers, while TCC are positive for these markers. TCC are also positive for CK 7 ^9^.

Elevated serum tumor markers, such as CA 125, CA 19-9 and CEA could be helpful in the diagnosis or follow-up, but they exceptionally elevated in PRMC and are not specific [13].

As for pre-operative imaging, ultrasound can aid in the initial evaluation of abdominal masses and can determine morphologic features suggestive of a malignant behavior of the tumor (thick wall, lobulation and presence of a solid component). However, CT scan and magnetic resonance imaging (MRI) are fundamental to determine the location, the extension and the content of the cyst. They can also show its impact on adjacent organs and detect possible signs of malignancy, such as ascites, peritoneal implants and lymphadenopathy. Analogous to our case, PRMC is usually unilocular with water-like fluid, thin-walled (<3 mm) and does not display papillary projections or mural nodules [8].

Due to the rarity of PRMC, other differential diagnoses should be considered when dealing with retroperitoneal cystic lesions. This includes benign tumors, such as cystic lymphangioma, cystic teratoma, cystic mesothelioma, tailgut cysts, epidermoid cysts and bronchogenic cysts; and other tumors with malignant behavior and cystic presentation, like neurilemmoma, paraganglioma and perianal mucinous carcinoma [14]. Interestingly, the radiologist, in our case, firstly misdiagnosed the tumor as a renal cyst; this is probably due to the lesion bordering the inferior pole of the kidney and being a far more frequent lesion. Therefore, a proper knowledge of this entity is fundamental, as to avoid a misdiagnosis that could lead to over-treating a benign lesion.

Since the benign or malignant nature of cystic retroperitoneal lesions can only be confirmed by pathology, a careful and complete surgical removal of the tumor is necessary; and most importantly, without rupture of the cyst, as to prevent possible tumor dissemination, following content spillage. Although the surgical approach, undertaken in our case, was successful, CT-guided percutanous drainage is not without risks, mainly fistula formation, bleeding and sepsis [15]. In the vast majority of the previous cases, the opted surgical approach for retroperitoneal cysts was a laparotomy, as to ensure a complete resection and a thorough per-operative evaluation. However, in certain instances, minimally invasive resection through laparoscopy have also been performed [16,17].

## 4 Conclusion

Primary retroperitoneal mucinous cystadenoma (PRMC) is a rare benign epithelial tumor that should be taken into account when confronted to retroperitoneal cystic lesions. A preoperative diagnosis, by CT and MRI, is essential, seeing that most of retroperitoneal tumors are malignant and that surgical management is different; which in the case of PRMC is more conservative and tends to be minimally invasive. The final diagnosis relies upon histological examination.

## Funding

None, The authors have received no funding from any agency in the public, private, or not-for-profit sectors.

## Provenance and peer review

Not commissioned, externally peer-reviewed.

## Ethical approval

N/A.

## Please state any sources of funding for your research

None, The authors have received no funding from any agency in the public, private, or not-for-profit sectors.

## Author contribution

SF: Writing-original, draft preparation, resources, review and editing.

SBH: Writing-review and editing, pathologic images, resources.

SB: Review and editing, radiologic images, resources.

AZ: Conceptualization, reviewing and editing, resources, supervision.

NL, RH, AB and CC: Reviewing and editing, resources.

All authors contributed to the development of the manuscript. All authors approved the final manuscript.

## Registration of research studies

N/A.

## Guarantor

Dr Safouane Frini.

## Consent

Written informed consent was obtained from the patient for publication of this case report and accompanying images. A copy of the written consent is available for review by the Editor-in-Chief of this journal on request.

## Declaration of competing interest

The authors declare that there are no conflicts of interest in this work.
